# Logarithms and exponentiation in MUMPS: routines for the clinical biochemistry laboratory computer system

**DOI:** 10.1155/S1463924685000268

**Published:** 1985

**Authors:** T. G. Pellar, A. R. Henderson

**Affiliations:** Department of Clinical Biochemistry University Hospital P. O. Box 5339, Postal Stn. A Ontario London N6A 5A5 Canada


Journal of Automatic Chemistry/*Journal of Clinical Laboratory Automation*, Vol. 7, No. 3 (Jul–Sep 1985), pp. 124–129

### Logarithms and exponentiation in MUMPS: routines for the clinical biochemistry laboratory computer system

**T. G. Pellar and A. R. Henderson**

*Department of Clinical Biochemistry, University Hospital, P.O. Box 5339, Postal Stn. A, London, Ontario, Canada N6A 5A5*

### Introduction

The BASIC language has a wide range of arithmetic functions such as natural and common logarithms, natural antilogarithm (exponential) and square root [1]. In addition, exponentiation is an available arithmetic operator. These features make BASIC a useful language for scientific work. By contrast, the very powerful data-base language MUMPS [2 and 3] lacks these imbedded arithmetic functions and operator, thus severely limiting the occasional use of this language in many scientific applications. For example, body surface area ($m^2$) is calculated by the expression:

$$W^{0{\cdot}425} \cdot H^{0{\cdot}725} \cdot 0{\cdot}007184$$

where $W$ = weight (kg) and $H$ = height (cm) [4]. This expression cannot currently be evaluated using Standard MUMPS. This is often inconvenient and alternative procedures are required to handle these requirements.

We have written two routines to generate common logarithms and antilogarithms thus allowing scientific functions such as exponentiations and root (including square root) calculations. These may be regarded as supplementing our CALCULATOR program [5], which allows a laboratory MUMPS system to be used as a simple calculator.

### Materials and methods

The University Hospital laboratory computer system is a 32-bit Data General Eclipse MV/6000 with 1 megabyte of core memory (Data General [Canada] Ltd, Mississauga, Ontario, Canada). The XL-87H video display terminal (Cybernex Ltd, Ottawa, Ontario, Canada) was used; this emulates a Hazeltine terminal. The computer operating and programming system was written in the MIIS dialect of MUMPS (Medical Information Technology, Inc., Cambridge, Massachusetts, USA) [6].

The operating system allows direct access to the MUMPS programming mode by means of passwords. Operating in this direct mode does not interfere with the main laboratory system or its data-base.

Instead of using standard series functions to represent logarithmic and exponential functions [7] two rational functions as described by Hastings were used [8]. These were, for $\log_{10} x$ over the range $1 \leqq x \leqq 10$:

$$\tfrac{1}{2} + C_1\left(\frac{x-\sqrt{10}}{x+\sqrt{10}}\right) + C_3\left(\frac{x-\sqrt{10}}{x+\sqrt{10}}\right)^3 + \ldots + C_9\left(\frac{x-\sqrt{10}}{x+\sqrt{10}}\right)^9,$$

where
$C_1$ = 0·868 591 718,
$C_3$ = 0·289 335 524,
$C_5$ = 0·177 522 071,
$C_7$ = 0·094 376 476,
$C_9$ = 0·191 337 714, and
$\sqrt{10}$ = 3·162 277 660 16837, and, for $10^x$, over the range $0 \leq x \leq 1$:

$$(10^x) = [1 + a_1x + a_2x^2 + \ldots\ldots a_7x^7]^2$$

where

$a_1$ = 1·151 292 77603,

$a_2$ = 0·662 730 88429,

$a_3$ = 0·254 393 57484,

$a_4$ = 0·072 951 73666,

$a_5$ = 0·017 421 11988,

$a_6$ = 0·002 554 91796, and

$a_7$ = 0·000 932 64267.

Both functions have periodic error curves of extremely low magnitude [8].

### The program

On calling the Log Functions Routine, the operator is asked to select from:

LOG
ANTILOG
EXPONENTIATION
ROOT
BODY SURFACE AREA
HELP.

The options are selected by entering the first letter of the required option. The HELP routine is shown in table 1. This narrative gives examples of the type of available calculation; advice is also provided regarding the decimal place option.

The LOG option returns the logarithm of a number to the selected decimal place; a decimal place entry greater than 15 forces the prompt 'Whole numbers 0 to 15 only'.

*Table 1. The HELP routine. This narrative outlines the use of each option. Each section is displayed sequentially on the screen by pressing <ENTER>.*

*Decimals*

At the beginning of each option, the user is asked to set the number of decimal places required in the answer. The default response is four; any response from 0 to 15 is valid.

Note that the system is only capable of handling 16 significant figures. Numbers requiring more than 16 digits will be padded with place-holding zeros to fit the requested format. Consider the following example of exponentiation for which six significant decimals were requested.

BASE: 486·23 EXPONENT: 7

486·23 RAISED TO THE POWER 7 IS 6425282938784834000·000000

In the answer, the first 16 digits are arithmetically significant with the following zeros present as place holders and format fulfillers.

*Log*

In this routine, a number for which the log is to be determined is input (underlined), and the calculated log is returned. The logarithm of a fraction is a negative number which may be represented in one of two ways. It may be expressed with a positive mantissa and a negative characteristic or with a negative mantissa and a negative characteristic. The first format is typically expressed with a bar over the characteristic; this program will express the bar format by preceding the characteristic with an underscore. The all negative format is written with a preceding negative and will be shown in brackets.

e.g.,
HOW MANY DECIMALS? 4/ /
NUMBER: 5·5 LOG OF 5·5 IS ·7404
NUMBER: 10·05 LOG OF 10·05 IS 1·0022
NUMBER: ·0098 LOG OF ·0098 IS __3·9911 (−2·0089)

*Antilog*

The antilog routine accepts the logarithmic value for a number and then returns the value of that number. The log of a fraction may be input using either the bar notation or the log expressed as an all negative value. Note that a characteristic is required even if it is zero.

e.g.,
HOW MANY DECIMALS? 4/ /
LOG: __2·5200 ANTILOG OF __2·5200 IS ·0331
LOG: −1·4803 ANTILOG OF −1·4803 IS ·0331
LOG: 2·20 ANTILOG OF 2·20 IS 158·4893
LOG: 0·0154 ANTILOG OF 0·0154 IS 1·0361

*Exponentiation*

This routine will accept a number and an exponent and then calculate the number raised by the exponent.

e.g.,
HOW MANY DECIMALS? 4/ / 0
BASE: 5 EXPONENT: 5
5 RAISED TO THE POWER 5 is 3125
HOW MANY DECIMALS? 4/ /
BASE: 5 EXPONENT: 5·5
5 RAISED TO THE POWER 5·5 IS 6987·7008

*Root*

The root function does the reverse of the exponentiation function by determining the root value of a number.

e.g.,
HOW MANY DECIMALS? 4/ / 0
BASE: 121 ROOT: 2
121 ROOT 2 IS 11
HOW MANY DECIMALS? 4 / / 6
BASE: 145·45 ROOT: 2·13
145.45 ROOT 2·13 IS 10·359970

*Body surface area*

Body surface area (as square meters) is evaluated according to the formula 0·007184.W to power 0·425. H to power 0·725 where W(eight) is kg.

e.g.,
HEIGHT (cm): 125
WEIGHT (kg): 50

Body surface area in square meters is 1·25.

*Table 2. Introductory program. This routine sets up the Menu for calling the options.*

```
LOG#      ; INTRODUCTORY PROG LOG FUNCTIONS 250285 TGP
        K S CL(1)= .868591718",CL(3)=".289335524",CL(5)=".177522071",CL(7)=".0943
          76476",CL(9)=".191337714",R10="3.16227766016837"
        S CA(1)="1.15129277603",CA(2)=".66273088429",CA-
          (3)=".25439357484",CA(4)=".07295173666",CA(5)=".01742111988",CA(6)=".00255491796",CA(7)=".00093
          264267"
        W #! S BF="BAD FORMAT"
        W !?5 F I=1:1:69 W $C(96)
        F I=1:1:3 W ?5,$C(96),?75,$C(96),!
        W ?5,$C(96),?28,"L O G   F U N C T I O N S",?75,$C(96),!
        F I=1:1:3 W ?5,$C(96),?75,$C(96),!
        W ?5 F I=1:1:71 W $C(96)
        W !
        F I=1:1:3 W ?5,$C(96),?75,$C(96),!
        W ?5,$C(96),?30,"OPTIONS:",?75,$C(96),!
        W ?5,$C(96),?75,$C(96),!
        W ?5,$C(96),?30,"LOG",?75,$C(96),!
        W ?5,$C(96),?30,"ANTILOG",?75,$C(96),!
        W ?5,$C(96),?30,"EXPONENTIATION",?75,$C(96),!
        W ?5,$C(96),?30,"ROOT",?75,$C(96),!
        W ?5,$C(96),?30,"BODY SURFACE AREA",?75,$C(96),!
        W ?5,$C(96),?30,"HELP",?75,$C(96),!
        F I=1:1:2 W ?5,$C(96),?75,$C(96),!
        W ?5 F I=1:1:71 W $C(96)
LA      R !!,"OPTION: ",ANS:300 W:ANS="?" ! S CNT I 'ANS K (N,ZUS,V) Q
LC      S CNT=$U(CNT),L=$T(OPT+CNT) I 'L W:ANS'="?" *7, "NOT FOUND":3 G LA
        I ANS="?" W !?25,$P(L;2) G LC
        I $E($P(L;2),1,$L(ANS))=ANS W $E($P(L;2),$L(ANS)+1,$L($P(L;2))),!  X $P(L;3) G LA
        G LC
OPT      ;LIST OF LOG OPTIONS
         ;LOG;C TPLOG@LOG
         ;ANTILOG;C TPLOG@ANTI
         ;EXPONENTIATION;C TPLOG2@POWER
         ;ROOT;C TPLOG2@ROOT
         ;BODY SURFACE AREA;C TPLOG3
         ;HELP;C TPLOGH
         G LC
```

The logarithm of a number <1 is expressed in two different ways [7]: negative characteristic—positive mantissa (for example log 0·0002 is $\bar{4}$·3010) or negative characteristic—negative mantissa (for example log 0·0002 is −3·6990). Because '$\bar{4}$' is not available in our system we have used the nomenclature of '$\underline{4}$'. Note that the wholly negative logarithm (−3·6990 in the example above) is necessary for certain manipulations (such as calculating the power of a number) so that availability of both forms is necessary. The ANTILOG option is self-explanatory; either form of logarithm can be used.

The EXPONENTIATION option requests the base and exponent and then responds:

*x* RAISED TO THE POWER *y* IS NNNN.

The ROOTS option requests the base and root then responds:

*x* ROOT *y* IS NNNN.

Finally, the BODY SURFACE AREA calculation is self-explanatory.

The programs are listed in tables 2–5.

*Table 3. The preliminary log and antilog routines. This program interacts with the program listed in table 4.*

```

TPLOG   ;LOG–ANTILOG FUNCTIONS 050285 TGP

 LOG     K (*CL,*CA,R10,BF) D SF

 LOG1    K (*CL,*CA,R10,BF,SD,SR) R !!,“NUMBER: “,NU:300 Q:($M(NU*1)=0)!(‘NU) S PN=NU

          I NU’?1NN I NU’?1NN“.”1NN I NU’?“.”1NN I NU’?1NN“.” W BF:3 G LOG1

         C TPLOGS@FLOG

         W ”     LOG OF “,PN,” IS “,LOGP I LOG<O W ” ( ”,LOGN” ) ”

         G LOG1

 ANTI    K (*CL,*CA,R10,BF) D SF

 ANTI1   K (*CL,*CA,R10,BF,SR,SD) R !!,“LOG: ”,LOG:300 Q: ‘LOG S PL=LOG

          I LOG’?“–”1NN“.”1NN I LOG’?“_”1NN“.” 1NN I LOG‘?1NN“.”1NN W BF:3,G ANTI1

          I LOG<0 D CON

          I $E(LOG,1,1)=“_” S LOG=$E(LOG,2,99),LOG=$M(LOG*–1)

         C TPLOGS@FANTI W “     ANTILOG OF ”,PL,“ IS ”,_“$M(”_ANTIL_“+”_SR_“,.”_SD_“)”

         G ANTI1

 CON      S LS=$E(LOG,1,($F(LOG,“.”)–2)),RS=$E(LOG,($F(LOG,“.”),99),RS=$M(RS*–1)

          S L=$M(LS–1),R=$M(RS+1),LOG=L_R

         Q

 SF      R !!,“HOW MANY DECIMALS? 4/ / ”,SD:300

          I ‘SD S SD=4

          I (15<SD)!(0>SD)!(SD’?1NN) W “WHOLE NUMBERS 0 TO 15 ONLY”:3 G SF

         F I=1:1:SD S SR=SR_0

          S SR=“.”_SR_5 Q

```

*Table 4. Log and antilog calculations.*

```

 TPLOGS  ; FIND LOG AND ANTILOG 050385 TGP

 FLOG     I NU>10 D SUB1

          I NU<1 D SUB2 S SIGN=“–”,K=LL

          S N1=$M(NU–R10),NU1=$M(NU+R10),N1=$M(N1/NU1)

          S NL(1)=N1

          F IL=1:1:8 S X=IL+1 S NL(X)=$M(NL(IL)*N1)

          F IL=1:2:9 S AAL(IL)=$M(NL(IL)*CL(IL))

          F IL=1:2:9 S TOTL=$M(AAL(IL)+TOTL)

          S TOTL=$M(TOTL+.5) S LOG=SIGN_K_TOTL I $E(LOG,1,1)=“.” S LOG=“0”_LOG

          S LOGP=LOG

          S _“LOGP=”_“$M(”_LOGP_“+”_SR_“,.”_SD_“)” I $E(LOGP,1,1)=“.” S LOGP=“0”_LOGP

          I LOGP<0 S LOGN=LOGP,LOGP=$M(LOGP*–1),LOGP=“_”_LOGP

          I LOGN S CH=$E(LOGN,1,($F(LOGN,“.”)–2)),MA=$E(LOGN,($F(LOGN,“.”)–1),99),LOGN=$M(CH+MA)

            I $E(LOGN,1,2)=“–.” S LOGN=$M(LOGN*–1),LOGN=“–0”_LOGN

         Q

 FANTI    I LOG<0 D SUB3

          I LOG>0 D SUB4

          S NA(1)=ALOG

          F IA=1:1:6 S X=IA+1 S NA(X)=$M(NA(IA)*ALOG)

          F IA=1:1:7 S AA(IA)=$M(NA(IA)*CA(IA))

          F IA=1:1:7 S TOTA=$M(AA(IA)+TOTA)

          S TOTA=$M(TOTA+1),TOTA=$M(TOTA*TOTA)

          S ANTIL=$M(TOTA*FACTOR)

            Q

 SUB1     F K=1:1 S NU=$M(NU*.1) Q:NU<10

            Q

 SUB2     F LL=1:1 S NU=$M(NU*10) Q:NU>1

         Q

 SUB3     S FACT=$E(LOG,2,($F(LOG,“.”)–2)),FACTOR=1 F M=1:1:FACT S FACTOR=$M(FACTOR*.1)

          S ALOG=$E)(LOG,($F(LOG,“.”)–1),99)

         Q

 SUB4     S FACT=$E(LOG,1,($F(LOG,“.”)–2)),FACTOR=1 F N=1:1:FACT S FACTOR=$M(FACTOR*10)

          S ALOG=$E(LOG,($F(LOG,“.”)–99)

         Q

```

### Program validation

These routines have been tested in a number of ways. First a complete table of four-figure logarithms was produced and compared with a set of commercially available tables [8]; the values were identical. Next a set of one- to eight-digit numbers were converted to their logarithms using 15 significant decimal places, their antilogarithms were formed and the recovered number compared with the original. Some examples are:

(1) log 2 = 0·301 030 126 730 358
antilog(log 2) = 2·000 000 612 526 983.

*Table 5. Power and root calculations. This program interacts with the program listed in table 4. This table also contains the body surface area calculations.*

```

TPLOG2   ; EXPONENTIATION–ROOT FUNCTIONS 050385 TGP

POWER  K (*CL,*CA,R10,BF) D SF

       R !!,"BASE: ",NU:300 Q:($M(NU*1)=0)!('NU) S G=NU

       I NU'?1NN I NU'?1NN"."1NN I NU'?"."1NN I NU'?1NN"." W BF:3 G POWER

POWER1 R " EXPONENT: ",EXP:300 Q:($M(EXP*1)=0)!('EXP)

       I EXP?1NN D SUB5 G POWER

       I EXP'?1NN I EXP'?1NN"."1NN I EXP'?"."1NN I EXP'?1NN"." W BF:3 G POWER1

       C TPLOGS@FLOG I LOG<0 D SUB6 C TPLOGS@FANTI

       E2 S LOG=$M(LOG*EXP) C TPLOGS@FANTI

       W !!,G," RAISED TO THE POWER ",EXP," IS ",_"$M("_ANTIL_"+"_SR_",."_SD_")" G POWER

ROOT   K (*CL,*CA,R10,BF) D SF

       R !!,"BASE: ",NU:300 Q:($M(NU*1)=0)!('NU) S G=NU

       I NU'?1NN I NU'?1NN"."1NN I NU'?"."1NN I NU'?1NN"." W BF:3 G ROOT

ROOT1  R "    ROOT: ",ROOT:300 Q:($M(ROOT*1)=0)!('ROOT)

       I ROOT'?1NN I ROOT'?1NN"."1NN I ROOT'?"."1NN I ROOT'?1NN"." W BF:3 G ROOT1

       C TPLOGS@FLOG I LOG<0 D SUB7 C TPLOGS@FANTI

       E  S LOG=$M(LOG/ROOT) C TPLOGS@FANTI

       W !!,G," ROOT ",ROOT," IS ",_"$M("_ANTIL_"+"_SR_",."_SD_")" G ROOT

SUB5   S ANS=1 F B=1:1:EXP S ANS=$M(ANS*NU)

       W !!,G," RAISED TO THE POWER ",EXP," IS ",_"$M("_ANS_"+"_SR_",."_SD_")" Q

SUB6   S CH=$E(LOG,1,($F(LOG,".")-2)),MA=$E(LOG,($F(LOG,".")-1),99)

       S CHA=$M(CH*EXP),MAN=$M(MA*EXP)

       S RES=$M(CHA+MAN)

       S LS=$E(RES,1,($F(RES,".")-2)),RS=$E(RES,($F(RES,".")-1), 99),RS="-"_RS

       S L=$M(LS-1),R=$M(RS+1),LOG=L_R

       Q

SUB7   S CH=$E(LOG,1,($F(LOG,".")-2)),MA=$E(LOG,($F(LOG,".")-1),99)

       S CHA=$M(CH/ROOT),MAN=$M(MA/ROOT)

       S RES=$M(CHA+MAN)

       S LS=$E(RES,1,($F(RES,".")-2)),RS=$E(RES,($F(RES,",")-1),99),RS="-"_RS

       S L=$M(LS-1),R=$M(RS+1),LOG=L_R

       Q

SF     R !!,"HOW MANY DECIMALS? 4 / / ",SD:300

       I 'SD S SD=4

       I (15<SD)!(0>SD)!(SD'?1NN) W "WHOLE NUMBERS 0 TO 15 ONLY":3 G SF

       F I=1:1:SD S SR=SR_0

       S SR="."_SR_5 Q

TPLOG3   ; BODY SURFACE AREA CALCULATION 060385 TGP

       S SD="2",SR=".005"

       K (*CL,*CA,R10,BF,SD,SR)

HT     R !!,"HEIGHT (cm): ",NU Q:('NU)!($M(NU*1)=0)

       I NU'?1NN I NU'?1NN"."1NN W BF:3 G HT

       C TPLOGS@FLOG K (*CL,*CA,R10,BF,SD,SR,LOG) S LOG=$M(LOG*0.725) C TPLOGS@FANTI S

         HT=ANTIL

WT     K (*CL,*CA,R10,BF,SD,SR,HT)

       R !!,"WEIGHT (kg): ",NU Q:('NU)!($M(NU*1)=0)

       I NU'?1NN I NU'?1NN"."1NN W BF:3 G WT

       C TPLOGS@FLOG K (*CL,*CA,R10,BF,SD,SR,LOG,HT) S LOG=$M(LOG*0.425) C TPLOGS@FANTI S

         WT=ANTIL

       S AREA=$M(HT*WT),AREA=$M(AREA*.007184+.005,.2) I $E(AREA,1,1)="." S AREA="0"_AREA

       W !!,"SURFACE AREA IN SQUARE METERS IS ",AREA

       G TPLOG3 Q

```

(2) input value 9437.0 recovered value 9437·0.
(3) input value 2288·0677 recovered value 2288·0679.
(4) input value 0·0005950 recovered value 0·0005950.

The largest error (of the order of 0·000 05%) occurs when mixed (i.e. integer and decimal) numbers are used. If the number is either an entire integer or decimal, the error is smaller (for example log 2 example given above).

Finally a set of one- to eight-digit numbers were exponentiated, their roots obtained and the recovered number compared with its original value. Using 15 significant decimal places in the calculation—as shown above for example 1—maximum errors of about 0·000 05% were obtained.

### Discussion

MUMPS routines have been provided for logarithms and exponentiation with defaults at four significant decimal places. If these defaults are over-ridden then the possibility of over- or under-flow in the arithmetic registers occurs. As Nonweiler warns [10], one must neither assume that computers are inevitably accurate, nor ignore the need for numerate or algebraic skills. However, if these routines are used sensibly they can be relied upon to be accurate at the level required in clinical biochemistry laboratories.

Program details for the HELP routine may be obtained by writing to the authors.

### References

1. Lien, D. A., *The BASIC Handbook,* 2nd edn (Compusoft Publishing, San Diego, 1981), 123, 190, 192 and 409.
2. Sherertz, D., *ANS MUMPS Programmers' Reference Manual 1983* (MUMPS Users' Group, College Park, 1983), 143.
3. Walters, R. F., Bowie, J. and Wilcox, J. C., *MUMPS Primer, Revised* (MUMPS Users' Group, College Park, 1982), 13.
4. Guyton, A. C., *Textbook of Medical Physiology,* 6th edn (Saunders Co., Philadelphia, 1981), 885.
5. Pellar, T. G., Rawal, N. and Henderson, A. R., *Journal of Automatic Chemistry,* **7** (1985), 95.
6. *MIIS Reference Manual*, Version S.MIIS.R-II-3.1 (Medical Information Technology, Inc., Cambridge, 1983), 1.
7. Franklin, D. A. and Newman, G. B., *A Guide to Medical Mathematics* (Blackwell Scientific Publications, Oxford, 1973), 61 and 96.
8. Hastings, C., *Approximations for Digital Computers* (Princeton University Press, Princeton, 1955), 128 and 144.
9. Nelson, R. D., *The Penguin Book of Mathematical and Statistical Tables* (Penguin Books, Harmondsworth, 1980), 14.
10. Nonweiler, T. R. F., *Computational Mathematics. An Introduction to Numerical Approximation* (Ellis Horwood Ltd, Chichester, 1984), 37.

### ANALYTICA 86

*3 to 6 June 1986 at the Munich Trade Fair Centre*

The scientific programme for this international meeting is divided into symposia, posters and the 'Analytica – Forum München' (in this latter sector, exhibiting companies will present papers on developments in industrial research). Topics to be covered include:

Separation methods
  Chromatographic methods, especially TLC/HPTLC, GC, LC/HPLC
  Electrophoretic methods
Combined methods: MS-MS, HPLC-MS, GC-MS, GC-IR, LC-MS, LC-NMR, FT-GC, GC-FTIR
Emission spectroscopy
Bioluminescence, chemiluminescence, fluorimetry
NMR, its application *in vivo*
Radiochemical procedures
Topochemical procedures
Enzymatic analysis
Cell and organ culture based analysis
Dry support reagents including stick tests
Progress in development of reference methods.

*Enquiries about the commercial exhibition and registration to Münchener Messe- und Ausstellungsgesellschaft mbH, Analytica 86, Postfach 12 10 09, D 8000 München 12, FR Germany. Information about scientific contributions from Professor Dr H. Feldmann, Inst. für Physiologische Chemie der Universität, Goethestrasse 33, D 8000 München 2, FR Germany.*

### FORTHCOMING PAPERS

Our No. 4 issue will include:

Automated preparation of biological samples prior to HPLC: Part 1—The use of dialysis for deproteinizing serum for amino-acid analysis; Part 2 – The combined use of dialysis and trace enrichment for analysing biological material
*J. D. H. Cooper and D. C. Turnell*

Haemoglobin analysis on whole blood by reflective photometry
*J. A. Lott and E. Khabbaza*

Laboratory information management – the CALS approach
*J. Boother*

